# The wood decay fungus *Cerrena unicolor* adjusts its metabolism to grow on various types of wood and light conditions

**DOI:** 10.1371/journal.pone.0211744

**Published:** 2019-02-05

**Authors:** Anna Pawlik, Marta Ruminowicz-Stefaniuk, Magdalena Frąc, Andrzej Mazur, Jerzy Wielbo, Grzegorz Janusz

**Affiliations:** 1 Department of Biochemistry, Maria Curie-Skłodowska University, Lublin, Poland; 2 Institute of Agrophysics, Polish Academy of Sciences, Lublin, Poland; 3 Department of Genetics and Microbiology, Maria Curie-Skłodowska University, Lublin, Poland; USDA Forest Service, UNITED STATES

## Abstract

*Cerrena unicolor* is a wood-degrading basidiomycete with ecological and biotechnological importance. Comprehensive Biolog-based analysis was performed to assess the metabolic capabilities and sensitivity to chemicals of *C*. *unicolor* FCL139 growing in various sawdust substrates and light conditions. The metabolic preferences of the fungus towards utilization of specific substrates were shown to be correlated with the sawdust medium applied for fungus growth and the light conditions. The highest catabolic activity of *C*. *unicolor* was observed after fungus precultivation on birch and ash sawdust media. The fungus growing in the dark showed the highest metabolic activity which was indicated by capacity to utilize a broad spectrum of compounds and the decomposition of 74/95 of the carbon sources. In all the culture light conditions, *p*-hydroxyphenylacetic acid was the most readily metabolized compound. The greatest tolerance to chemicals was also observed during *C*. *unicolor* growth in darkness. The fungus was the most sensitive to nitrogen compounds and antibiotics, but more resistant to chelators. Comparative analysis of *C*. *unicolor* and selected wood-decay fungi from different taxonomic and ecological groups revealed average catabolic activity of the fungus. However, *C*. *unicolor* showed outstanding capabilities to catabolize salicin and arbutin. The obtained picture of *C*. *unicolor* metabolism showed that the fungus abilities to decompose woody plant material are influenced by various environmental factors.

## Introduction

Wood degrading saprotrophic fungi have evolved unique biochemical pathways allowing them to assimilate a vast array of both simple and complex nutrients and to produce a variety of metabolites. Elucidating the mechanism of biological wood decay is ecologically important not only due to the role of fungi in the carbon cycle, but also due to its economic significance [1]. Both the environmental and economic roles of saprotrophic fungi arise from their metabolic versatility, which includes the production of a wide range of enzymes directly or indirectly linked to the degradation of organic residues [2]. Wood-destroying fungi that cause cell wall degradation can be classified according to the type of decay produced. The best-known types are brown rot, soft rot, and white rot. Each fungal type produces a different set of enzymes and is able to degrade different plant materials and thus colonize different ecological niches. Environmental factors greatly influence decay caused by brown rot and white rot fungi [3]. To sense light, only a few photoreceptor systems have developed during evolution. In fungi, photoreceptors controlling metabolism, developmental processes, and physiological adaptations as well as the circadian clock have already been found, suggesting that these organisms were able to sense light from blue to far red [4, 5].

*Cerrena unicolor* is a wood-degrading basidiomycete of the *Polyporaceae* family causing extensive white rot [6, 7]. It is also commonly known as a mossy maze polypore; however, it bears the general features of fungi belonging to the genus *Trametes* [8]. *Cerrena* is globally distributed in Europe, Africa, and South America, and is an aggressive wood-decay organism [7, 9]. This fungus is thought to be initially parasitic on living trees, becoming saprobic on the dead wood. *C*. *unicolor* is found year-round forming overlapping clusters on many deciduous hardwoods such as *Aesculus hippocastanum*, *Fraxinus excelsior*, *Acer* sp., *Betula* sp., *Fagus* sp., or *Quercus* sp., but is very rarely reported on conifers. Moreover, strains of *C*. *unicolor* are also well described as potential bioproducers of industrially relevant enzymes such as extracellular laccase, manganese peroxidase, versatile peroxidase [10–13], cellobiose dehydrogenase [14], xylanase, and cellulase [15]. It has also been demonstrated recently that *C*. *unicolor* can be a source of other bioactive compounds with pharmacological and medical importance [16, 17]. Recent RNAseq based transcriptomic analysis presented by Janusz et al. [18] has provided new insights into differentially expressed *C*. *unicolor* genes related to diverse metabolic pathways employed by the fungus in response to changing growth conditions. However, detailed phenotypic characterization allowing correlation between the genetic and metabolic aptitudes of the fungus has not been studied. In the context of *C*. *unicolor* abilities to degrade wood material and production of biotechnologically significant compounds, better understanding of selective substrate utilization may be extremely valuable. Furthermore, it can contribute to elucidation of the metabolic changes induced in the fungus cell by variable environmental conditions.

The emergence of high throughput Next-Generation Sequencing (NGS) techniques has accelerated the discovery of novel genes and has enabled further exploration and understanding the differential expression of decay-related gene families. Phylogenomic analyses of genomes of wood degrading fungi have provided insight into the diversity and evolution of the decay apparatus in basidiomycetes [19–21]. With the development of the Biolog Phenotype MicroArray (PM) technology, high throughput determination of microbial nutritional requirements and global phenotypic characterization became possible. With its measurement of microorganisms’ utilization capabilities against hundreds or thousands of different compounds, the PM system complements genome and proteome research. Thus, the PM system is suitable for studies of metabolism and gene expression [2, 22] providing a direct experimental linkage between an organism’s genotype and phenotype [22].

Here, a comparative metabolic analysis of *C*. *unicolor* FCL139 and selected wood-decay fungi belonging to different taxonomic and ecological groups was performed. The complete nutritional and chemical sensitivity profiles of white rot fungus *C*. *unicolor* FCL139 cultivated in variable light conditions and sawdust substrate using the Biolog FF (for Filamentous Fungi) plates and the PM system were also analyzed.

## Materials and methods

### Fungal strains and cultivation

Fungal strains (Table 1) representing different rot lifestyles (white and brown rot, respectively) and capable of selective or nonselective wood decomposition, deposited in the Fungal Culture Collection (FCL) of the Department of Biochemistry, Maria Curie–Sklodowska University, Lublin, Poland, were used in this study. The stock cultures were maintained on 4% (w/v) malt extract agar (Difco, BD, USA) slants. The slants were inoculated with mycelia and incubated at 28°C for 10 days and then used for seed culture inoculation. As an inoculum, ca. 5 mm^2^ of the slants were punched out with a sterilized cutter. Then the mycelia of each strain were transferred into a 100-ml liquid Lindeberg-Holm (LH) medium [23] in a 250-ml Erlenmayer flask. The seeds were cultivated in the dark at 28°C. Next, ten-day-old mycelia were homogenized in a disperser homogenizer T18 basic ULTRA-TURRAX (IKA, Staufen, Germany) and subjected to a selected Biolog procedure as described below.

**Table 1:** Fungal strain used in this study.

Strain number in FCL^a^	Strain name	Strain source	Taxonomic affiliation (order)	GenBank Accession to rRNA genes sequence	Strain description
FCL3	*Porodaedalea pini* *(Phellinus pini)*	MFP^e^	Hymenochaetales	KY474050	WR^f^, ‘red ring rot’ or ‘white speck’, selective
FCL7	*Trametes versicolor*	ATCC ^d^ 44308	Polyporales	KY474048	WR^f^, nonselective
FCL99	*Phlebia radiata*	ATCC ^d^ 64658	Polyporales	DQ056859	WR^f^, nonselective
FCL139	*Cerrena unicolor*	BIUR^b^	Polyporales	DQ056858	WR^f^, nonselective
FCL236	*Phanerodontia chrysosporium* *(Phanerochaete chrysosporium)*	FCTUA^c^	Polyporales	FJ594058	WR^f^, nonselective
FCL397	*Fomitopsis betulina (Piptoporus betulinus)*	environmental	Polyporales	KY474049	BR^g^, selective
FCL400	*Armillaria gallica*	environmental	Agaricales	KY474051	WR^f^ or ‘butt rot’, nonselective
FCL457	*Fistulina hepatica*	environmental	Agaricales	KY474052	BR^g^, selective

^a^FCL–Fungal Collection of Lublin, Lublin, Poland

^b^BIUR–Botany Institute II, Regensburg University, Regensburg, Germany

^c^FCTUA–Forest Products Chemistry Laboratory, University of Agriculture, Tokyo, Japan

^d^ATCC–American Type Culture Collection, USA

^e^MFP–Museum of Fungi, Paris, France

^f^WR–white rot

^g^BR–brown rot

### Comparative analysis of the fungal metabolic profile using Biolog FF MicroPlates (FF MPs)

The utilization of particular nutrients by each of the strains based on 95 carbon sources were evaluated using the Biolog FF MP (Biolog, Inc., Hayward, CA) containing tetrazolium dye. The inoculation procedure was based on the original FF MP (Biolog Inc., Hayward, CA) technique (supplied manufacturer’s protocol) and the protocol described elsewhere [24]. The suspension of the mycelia in inoculating fluid (FF-IF, Biolog) was adjusted to 75% of transmittance measured by a turbidimeter (Biolog). 100 μl of the above-mentioned mycelial suspension were added to each well and the inoculated microplates were incubated at 28°C in the OmniLog ID System (Biolog, Inc., Hayward, CA) in uncontrolled light conditions. The optical density was determined using a Biolog microplate reader for each plate at 24-h intervals over the period of 10 days at 490 nm (mitochondrial activity) and 750 nm (mycelial growth). The experiments were carried out in two biological replications for each strain. All measurements were performed in triplicates. The most consistent readings came from the 8-day old Biolog plates and these were used in the analyses.

Data from all experiments were combined in a single matrix and analyzed with the STATISTICA 9.0 (StatSoft, Inc., Tulsa, OK) software package. All data were subjected to descriptive statistical evaluations (mean, minimum, maximum, and standard deviation values) and checked for outliers. The average well color developments (AWCDs) of the different replicates were calculated, where AWCD equals to the sum of the difference between the OD of the blank well (water) and substrate wells divided by 95 (the number of substrate wells in the FF plates) developed by the fungus after 192 h of incubation. Functional diversity was measured as substrate richness. The number of different substrates utilized by the strain (counting all positive OD readings with the threshold ≥ 0.25) was calculated. Cluster analysis [25, 26] was performed to detect groups in the data set. In most cases, the cluster-joining analysis was made with Euclidian distance and complete linkages as the amalgamation rule, i.e., distances between clusters were determined by the greatest distance between any two objects in the different clusters. One-way or main-effect analyses of variance ANOVAs (confidence interval 95%) were performed to compare the growth of selected strains on individual carbon sources. ANOVA was followed by a post-hoc analysis using Tukey’s HSD (Honestly Significant Difference) test. The summed data matrices also were evaluated following multidimensional scaling to detect additional relationships between variables.

### Evaluation of the effect of *Cerrena unicolor* growth on various sawdust media on carbon and energy source utilization profiles using Biolog PM panels

Biolog PM1 and PM2 96-well microplates containing different carbon and energy sources were selected for in-depth phenotypic analysis of the correlation between growth on a specific sawdust substrate and utilization of particular nutrients by *Cerrena unicolor*. 190 substrates were included in the analysis and the procedure was as follows. 1.5% agar plates containing 10% of maple, ash, and birch sawdust and Lindeberg-Holm control medium [23] were inoculated with 2 ml of homogenized 10-day-old mycelia of *C*. *unicolor* as described above. Next, the plates were incubated in the dark at 28°C for ten days. Mycelial passaging through sawdust substrates were performed three times by punching out about 5 mm^2^ of the agar plate with a sterilized cutter. Inoculated plates were incubated in the dark at 28°C for ten days. Then, the cultivated mycelia were removed from the surface of agar plates by gentle rubbing across the surface with a sterile cell scraper and transferred into sterile inoculating fluid (FF-IF, Biolog). The suspension of the mycelia was adjusted to 62% of transmittance measured by a turbidimeter (Biolog). 100 μl of the above-mentioned mycelial suspension was added to each well and the inoculated microplates were incubated in stationary liquid growth conditions at 28°C in the OmniLog ID System (Biolog, Inc., Hayward, CA). Data were collected every 24 hours using Biolog Microstation (Biolog, Inc., Hayward, CA). The experiments were carried out in two biological replications for each sawdust substrate. The well color development was measured 3 times every 24 hours of incubation for 14 days, which provided both amplification and quantitation of the phenotype. The average values of color development from all readings for each compound recorded at 490 nm and 750 nm were used for data analysis. Analysis of variance (ANOVA) was used to determine the differences in the rate of utilization of a particular substrate in relation to the sawdust that was used. Post-hoc analyses were performed using Tukey’s test (HSD). All data were presented as 95% confidence intervals. Statistical significance was established at P < 0.05. Statistical analyses were performed using STATISTICA 10.0 (StatSoft, Inc., Tulsa, OK) software package.

### Evaluation of the effect of light conditions on *Cerrena unicolor* metabolic profiles and chemical sensitivity using Biolog FF and PM panels

The effect of different light conditions on *C*. *unicolor* metabolic abilities to utilize carbon substrates and various chemical agents was measured using Biolog FF MicroPlates (95 carbon sources) and Phenotype MicroArray chemical sensitivity panel PM21-PM25 (120 chemical compounds in four different concentrations). Cell suspensions were prepared from *C*. *unicolor* FCL139 mycelia cultured into a 100-ml liquid Lindeberg-Holm (LH) medium [23] in a 250 ml Erlenmayer flask. The mycelia were then cultivated at 28°C in incubators equipped with LED illumination cassettes (KT 115, Binder, Germany). Continuous light conditions (20 lux) were provided throughout the entire period of *C*. *unicolor* cultivation. The following light conditions were applied: white (color temperature 4000–4750 K), green (510–520 nm), blue (465–470 nm), and red (620–625 nm) light and dark as a control, respectively. Next, 10-day-old mycelia were homogenized in a disperser homogenizer T18 basic ULTRA-TURRAX (IKA, Staufen, Germany) and transferred into sterile inoculating fluid (FF-IF, Biolog). The turbidity of the suspension was unified and adjusted to 75% (FF) or 62% (PM) of transmittance, and then supplemented with yeast nitrogen base and D-glucose, according to the manufacturer’s protocol. The FF and PM21-PM25 plates were inoculated with the cell suspension (100 μl/well) and then incubated at 28°C for 10 days in an incubator providing appropriate light conditions as described above. The changes in the colour in the wells were measured every 24 hours, which provided both amplification and quantitation of the phenotype. Data collection and analysis were performed using the Biolog Microstation (Biolog, Inc., Hayward, CA) and Phenotype Microarray software (Biolog, Inc., Hayward, CA). The tetrazolium colour development as a function of time was determined at 490 nm (mitochondrial activity) and 750 nm (mycelial growth). All measurements were performed in triplicates in two biological replications. Metabolic and chemical sensitivity profiles were determined for each lighting treatment. All statistical analyses were calculated and performed as for the PM1 and PM2 plates.

## Results

### Comparative analysis of the metabolic profiles of *C*. *unicolor* and other wood-rotting fungi

**Fig 1:**
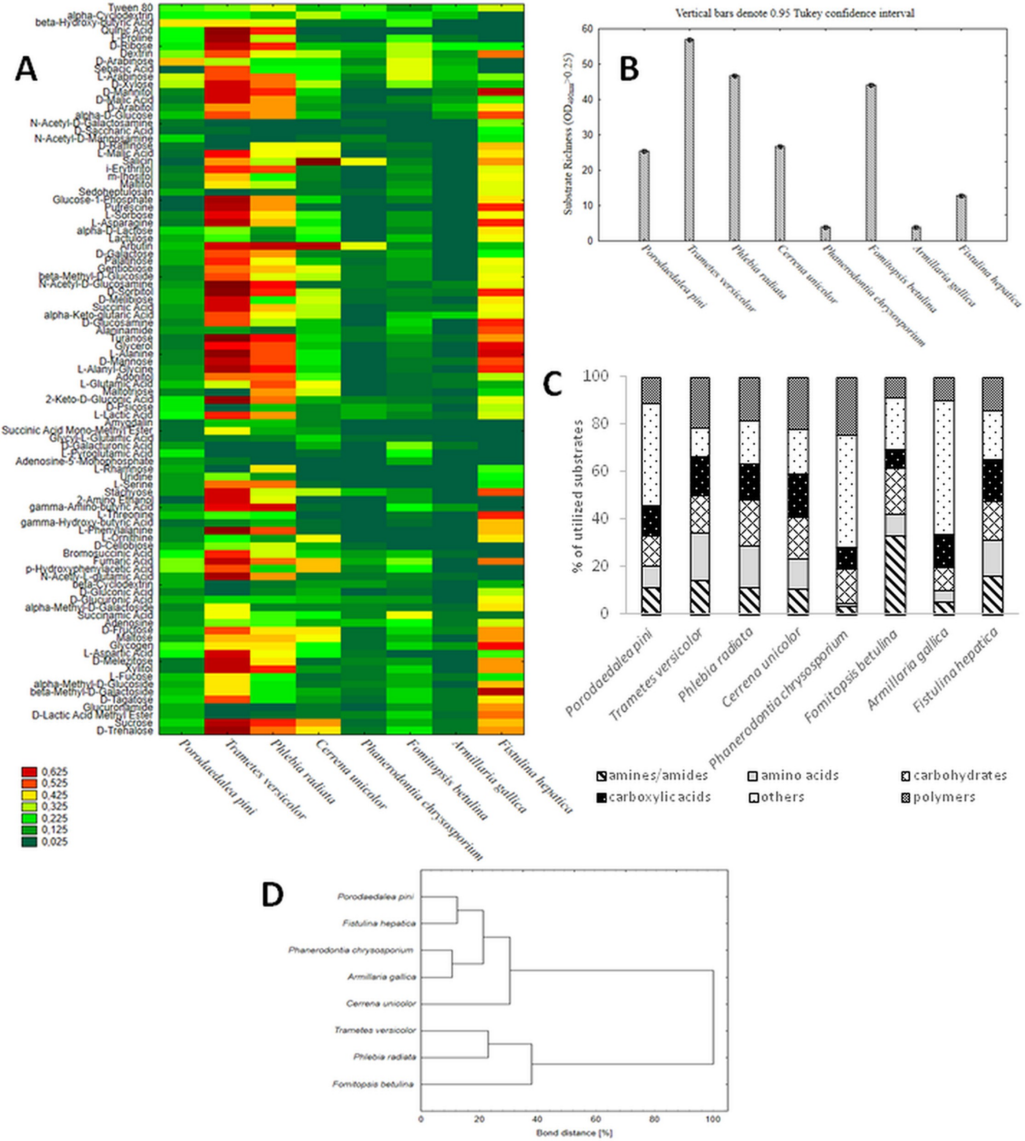
Comparison of metabolic aptitudes of different wood-rotting fungi. (A) Phenotype profiles of *P*. *pini* FCL3, *T*. *versicolor* FCL7, *P*. *radiata* FCL99, *C*. *unicolor* FCL139, *P*. *chrysosporium* FCL236, *F*. *betulina* FCL397, *A*. *gallica* FCL400, and *F*. *hepatica* FCL457 determined by Biolog FF MicroPlates during 192 hours of incubation with respective substrates. The color scale applied into the heat maps indicates the growth of the fungus (mitochondrial activity A_490nm_) on a particular substrate. (B) Metabolic diversity (substrate richness) of the fungi representing different rot lifestyles; vertical bars denote 0.95 confidence intervals. (C) Metabolic preferences of fungi towards utilization of a particular group of carbon substrates. (D) Cluster analysis-based dendrogram showing similarity of the FF MicroPlate C-sources utilization profiles of the analyzed strains; *P*. *pini* FCL3, *T*. *versicolor* FCL7, *P*. *radiata* FCL99, *C*. *unicolor* FCL139, *P*. *chrysosporium* FCL 236, *F*. *betulina* FCL397, *A*. *gallica* FCL400, and *F*. *hepatica* FCL457.

**Fig 2:**
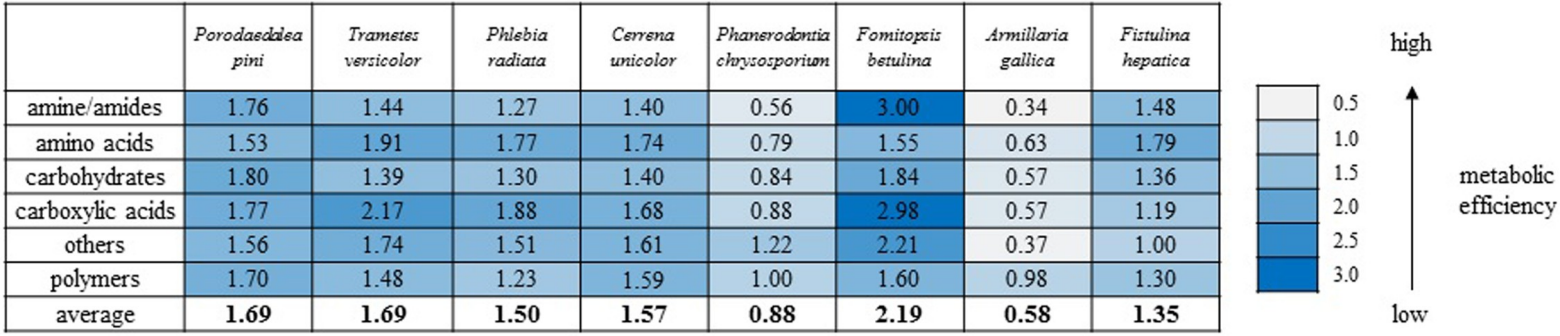
Ratio between normalized values of substrate use (OD 490nm) and biomass production (OD 750nm) defining theoretical metabolic efficiency of different wood-decaying fungi; *P*. *pini* FCL3, *T*. *versicolor* FCL7, *P*. *radiata* FCL99, *C*. *unicolor* FCL139, *P*. *chrysosporium* FCL 236, *F*. *betulina* FCL397, *A*. *gallica* FCL400, *F*. *hepatica* FCL457.

The use of the FF MicroPlates, AWCD analysis, and determination of mitochondrial activity allowed comparison of the metabolic diversity of fungi representing various rot lifestyles (Table 1). Their substrate utilization profiles revealed a great variability (Fig 1A), and the substrate richness (R) values, calculated as the number of utilized substrates, demonstrated significant differences (Fig 1B). It should be noted that FF plates should be considered as a strongly selective culturing media and if no/weak growth is observed it usually means that the tested compound cannot be utilized by the fungus and consequently cannot support its growth. *Trametes versicolor*, *Phlebia radiata*, and *Fomitopsis betulina* displayed the highest catabolic activities, which was reflected by their capabilities to assimilate 57 of 95 (which means assimilation of 60% of the total number of substrates), 46/95 (48%), and 44/95 (46%) of the total number of the substrates, respectively (Fig 1B). *Phanerodontia chrysosporium* and *Armillaria gallica* were able to utilize only 3 C-sources (3.1% on average). *C*. *unicolor* and *Fistulina hepatica* metabolized a comparable number of substrates 25-26/95 (26–27%), while *Porodaedalea pini* metabolized 12 carbon compounds (16.6%), indicating the average abilities of utilization of C-sources. Qualitative analysis of the proportions between the particular groups of utilized substrates indicated that *C*. *unicolor* was capable of metabolizing a defined group of compounds in equal proportions, similar to *T*. *versicolor*, *P*. *radiata*, and *F*. *hepatica*, while other strains exhibited some metabolic preferences for certain categories of C-substrates (Fig 1C). *P*. *pini*, *P*. *chrysosporium*, and *A*. *gallica* preferentially utilized various polymers, while amides/amines were frequently degraded by *F*. *betulina*. However, there was no clear correlation between the fungal lifestyle and carbon substrate utilization preferences and their overall metabolic profiles. In general, carbohydrates were used most universally by all the strains, while carboxylic acids proved to be the least preferred as a C-source (Fig 1A). *C*. *unicolor* showed outstanding capabilities to catabolize salicin and arbutin. Taken together, the results presented above allowed grouping the tested strains into two major clusters (A and B) (Fig 1D). Strains classified as extensive metabolizers (*T*. *versicolor*, *P*. *radiata*, and *F*. *betulina*) constituted group B. The cluster denoted with A comprised strains displaying weak (*P*. *chrysosporium*, *A*. *gallica*) and moderate (*P*. *pini*, *F*. *hepatica*) abilities to metabolize the defined groups of substrates, which were arranged in individual subclusters, with *C*. *unicolor* also representing “medium metabolizers” forming a separated branch of the dendrogram. Additionally, the ratios between normalized values of mitochondrial respiration (OD490nm) and fungal growth (OD750nm) calculated for the different groups of substrates showed diverse metabolic efficiency of the fungi (Fig 2).

### Evaluation of the effect of *Cerrena unicolor* growth on different sawdust substrates on carbon and energy source utilization profiles

**Fig 3:**
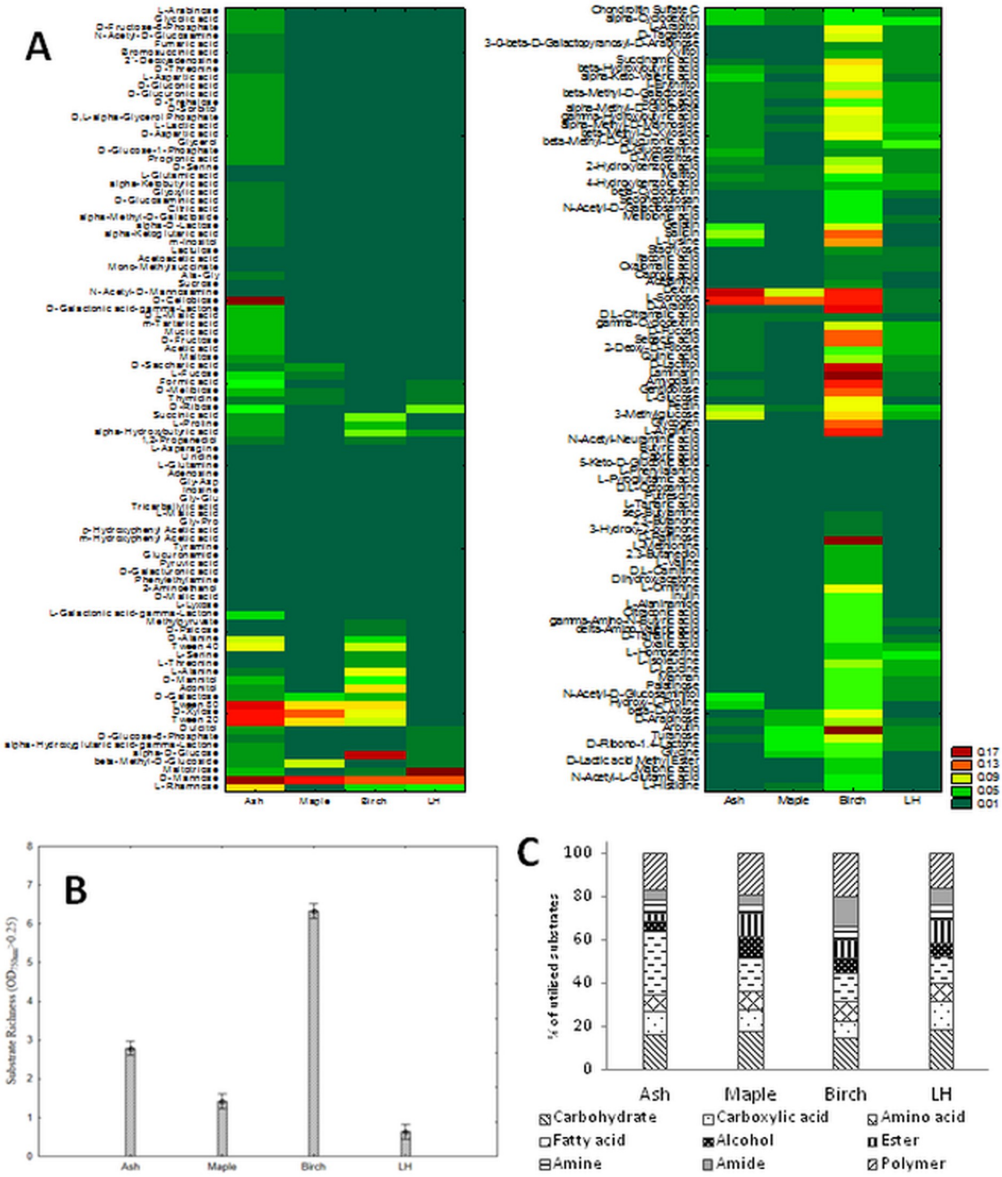
Utilization profiles of *C*. *unicolor* grown on different sawdust substrates. (A) Phenotype profiles of *C*. *unicolor* FCL139 obtained with PM1 and PM2 panels after fungus precultivation on ash, maple, and birch sawdust and LH control medium. The color scale applied into the heat maps indicates the growth of the fungus (OD 750 nm) on a particular substrate. (B) Substrate richness values of *C*. *unicolor* FCL139 precultured on ash, maple, and birch sawdust and LH mineral medium; vertical bars denote 0.95 confidence intervals. (C) Metabolic preferences of *C*. *unicolor* FCL139 towards particular carbon and energy sources observed after fungus precultivation in media containing different sawdust substrates and LH control medium.

To reveal the effect of growth conditions of *C*. *unicolor* on its metabolism, the carbon and energy utilization profile of the fungus after its growth on sawdust-containing media was examined. After precultivation in SSF (solid-state fermentation) conditions on birch, ash, and maple sawdust and the LH control medium, the metabolic profiles of *C*. *unicolor* FCL139 showed significant diversity in fungus metabolism between the compared culture treatments. It should be noted that during 14 days of *C*. *unicolor* incubation on selective media (PM plates) only mycelium was formed and no other morphological structures were observed in this cultivation conditions. The highest catabolic activity of *C*. *unicolor* was observed after fungus precultivation on birch and ash sawdust media, whereas the use of the control (LH) medium resulted in the weakest biomass production (Fig 3A). Similarly, fungal preculture on birch and ash sawdust media resulted in the highest substrate richness values (Fig 3B). In the studied conditions, there was a clear bias in fungus metabolic preferences towards a particular group of substrates (Fig 3C). *C*. *unicolor* cultivated on the ash sawdust exhibited preferences for utilization of fatty acids (29%), while polymers and carbohydrates were frequently metabolized carbon sources after *C*. *unicolor* growth on birch (20%) and maple (19%) sawdust and in the control medium (18%), respectively. After fungal growth on the birch medium, amides were more often utilized in comparison to the other preculture conditions (Fig 3C). In general, amines appeared to be the least frequently utilized group of substrates (5.97% on average) and polymers were the most favorable sources utilized by *C*. *unicolor* (18.13% on average). The analyses of individual compounds metabolized by the fungus showed that carbohydrates represented a group of compounds that were most effectively and universally used by *C*. *unicolor* in all the sawdust culture conditions. More specifically, mannose, sorbose, and xylose were usually found among those carbohydrates. On the other hand, cellobiose and maltotriose were metabolized extensively by the fungus only after the ash and LH precultivation, respectively. Instead, lactitiol, raffinose, glucose, amygdalin, arabitol, and arbutin were used almost exclusively by *C*. *unicolor* in the birch culture condition (Fig 3A). Polymers constituted the second group of compounds effectively degraded by *C*. *unicolor*. Dextrin, pectin, laminarin, and glycogen were extensively utilized during fungus growth in the individual culture treatments and dextrin and pectin turned out to be universal polymeric carbon sources. Surprisingly, the fungus demonstrated the ability to utilize all the tested polymeric substrates only after *C*. *unicolor* growth on the birch sawdust medium (Fig 3A). Strain FCL139 showed moderate capabilities of amino acids utilization but, similarly to the polymeric compounds, it was able to grow using most of the amino acids only after mycelia precultivation on the birch sawdust medium. The highest growth rate of the fungus was observed when arginine, lysine, and ornithine were the sources of energy (Fig 3A).

### Effect of light conditions on *C*. *unicolor* metabolic profiles

**Fig 4:**
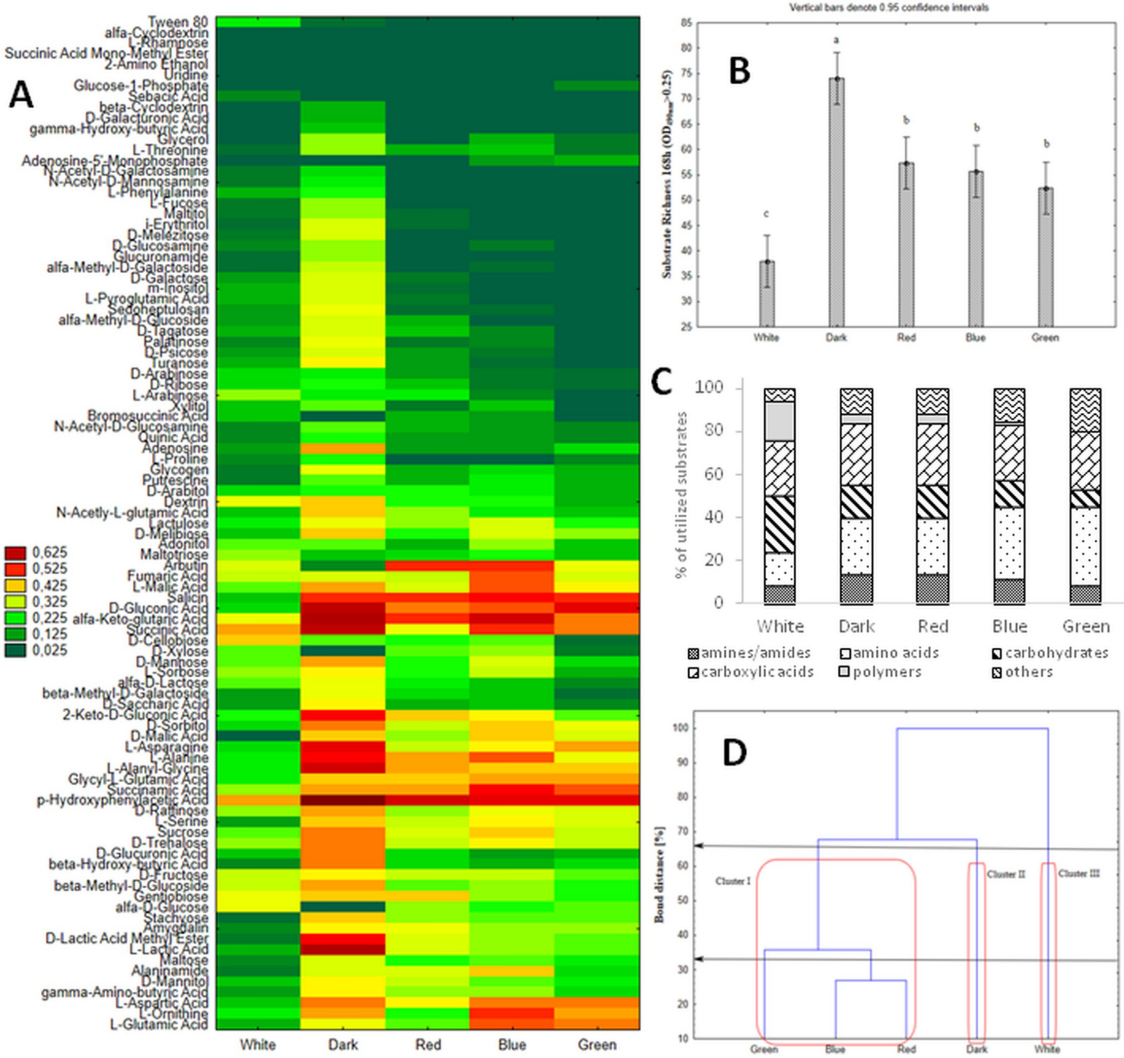
The effect of light on *C*. *unicolor* metabolic profiles. (A) FF MP substrate utilization profile of *C*. *unicolor* FCL139 cultured in different light conditions (white, red, blue and, green light, darkness) during 168 hours of incubation. The color scale applied into the heat maps indicates the growth of the fungus (mitochondrial activity A_490nm_) on a particular substrate. (B) Substrate richness (R) values of *C*. *unicolor* FCL139 cultured in different light conditions (white, red, blue and, green light, darkness); vertical bars denote 0.95 confidence intervals. (C) Metabolic preferences of *C*. *unicolor* FCL139 cultured in different light conditions (white, red, blue and, green light, darkness) towards utilization of a particular group of carbon substrates. (D) Cluster analysis-based dendrogram showing similarity of FF MicroPlates C-source utilization profile of *C*. *unicolor* FCL139 cultured in different light conditions (white, red, blue and, green light, darkness).

**Fig 5:**
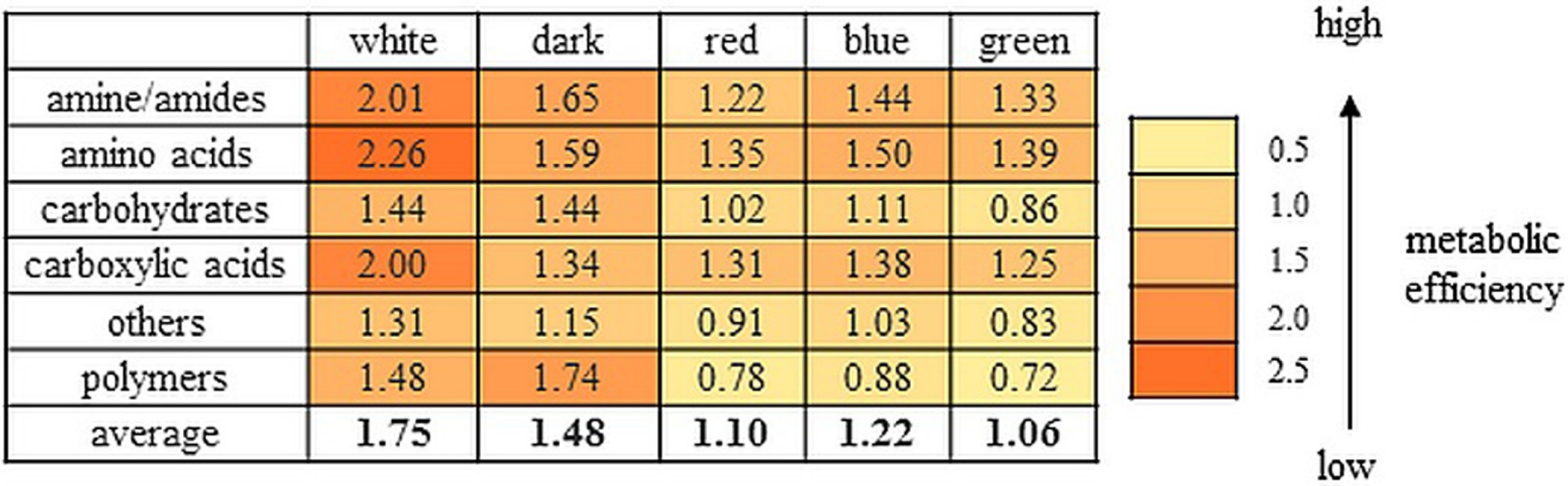
Ratio between normalized values of substrate use (OD 490nm) and biomass production (OD 750nm) defining theoretical metabolic efficiency of *C*. *unicolor* cultured in different lightning treatment (white, red, blue and, green light, darkness).

Next, the influence of various light conditions (20 lux, including white, red, blue, green light, as well as darkness) on *C*. *unicolor* metabolic profiles was evaluated using FF MPs and PM panels. The *C*. *unicolor* substrate utilization profiles varied greatly during fungus growth in the lighting treatments specified above (Fig 4A), which was reflected by the index of overall metabolic activity (AWCD). Interestingly, in the darkness, *C*. *unicolor* was not able to utilize D-glucose, D-xylose, and bromosuccinic acid, which were efficiently metabolized in other light conditions. Moreover, sebacic acid was exclusively utilized by the fungus grown in the white light, similarly to Tween 80 (limited utilization of this compound was demonstrated in the darkness). In all the culture conditions, *p*-hydroxyphenylacetic acid was the most readily metabolized compound (Fig 4A). Significant differences were demonstrated in the R values, as calculated for the most consistent and distinguishing readings (7-day-old FF MPs). The darkness applied for *C*. *unicolor* growth resulted in the highest catabolic activity of the fungus, which was indicated by the decomposition of 74/95 of the total number of the tested C-sources. In turn, during the white light cultivation, the fungus assimilated only 38 substrates, i.e. 40% of total number (Fig 4B). The metabolic preferences of the *C*. *unicolor* for a particular group of substrates were shown to be correlated with the light conditions. The capabilities to utilize polymers and carbohydrates decreased in the following manner white>dark = red>blue>green lights, while the use of amino acids was changing in the opposite direction. The level of carboxylic acids utilization was comparable in each of the tested conditions (Fig 4C). The clustering analysis allowed grouping the *C*. *unicolor* metabolic profiles obtained in different light conditions into three separate clusters (Fig 4D). The first cluster (I) comprised substrate utilization profiles of the fungus grown in the green, blue, and red lights, while those grown in the darkness and especially in the white light culture conditions formed distinct groups, i.e. cluster II and III, respectively. The clustering analysis confirmed the light-dependent metabolic diversity of *C*. *unicolor* and emphasized substantial distinction of the fungus substrate utilization profile in the white light culture treatment. Additionally, the ratios between the normalized values of mitochondrial activity (OD490nm) and biomass production (OD750nm) of *C*. *unicolor* cultured in different light conditions also showed diverse metabolic efficiency of the fungus (Fig 5).

### Effect of light conditions on *C*. *unicolor* chemical sensitivity

**Fig 6:**
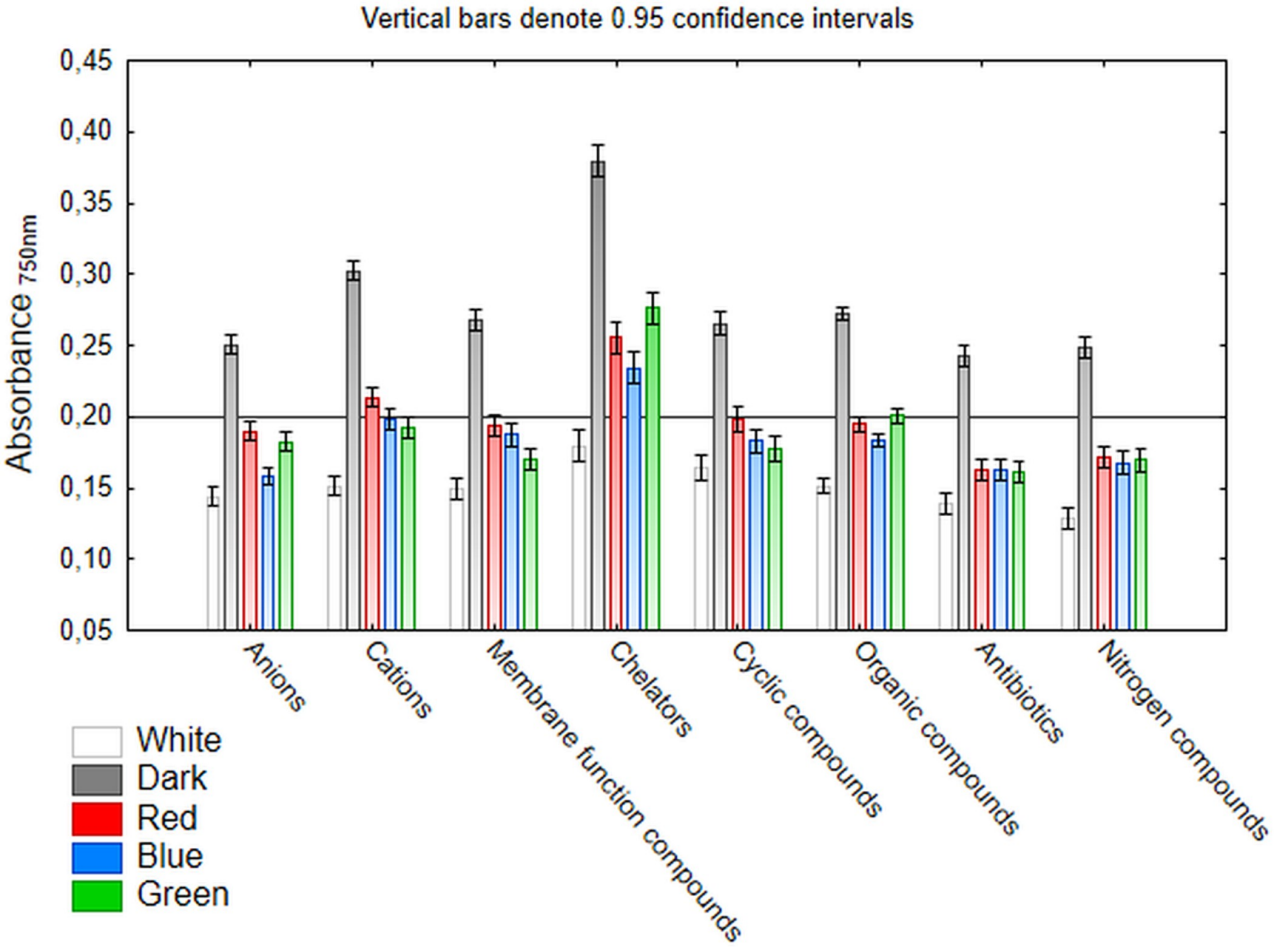
*C*. *unicolor* growth in different lighting treatments (white, red, blue and, green light, darkness) in the presence of various groups of potentially toxic chemical compounds. The scale represents growth values (OD 750 nm) after 10 days of incubation. The horizontal line at a value of 0.2 represents the growth threshold considered positive.

**Fig 7:**
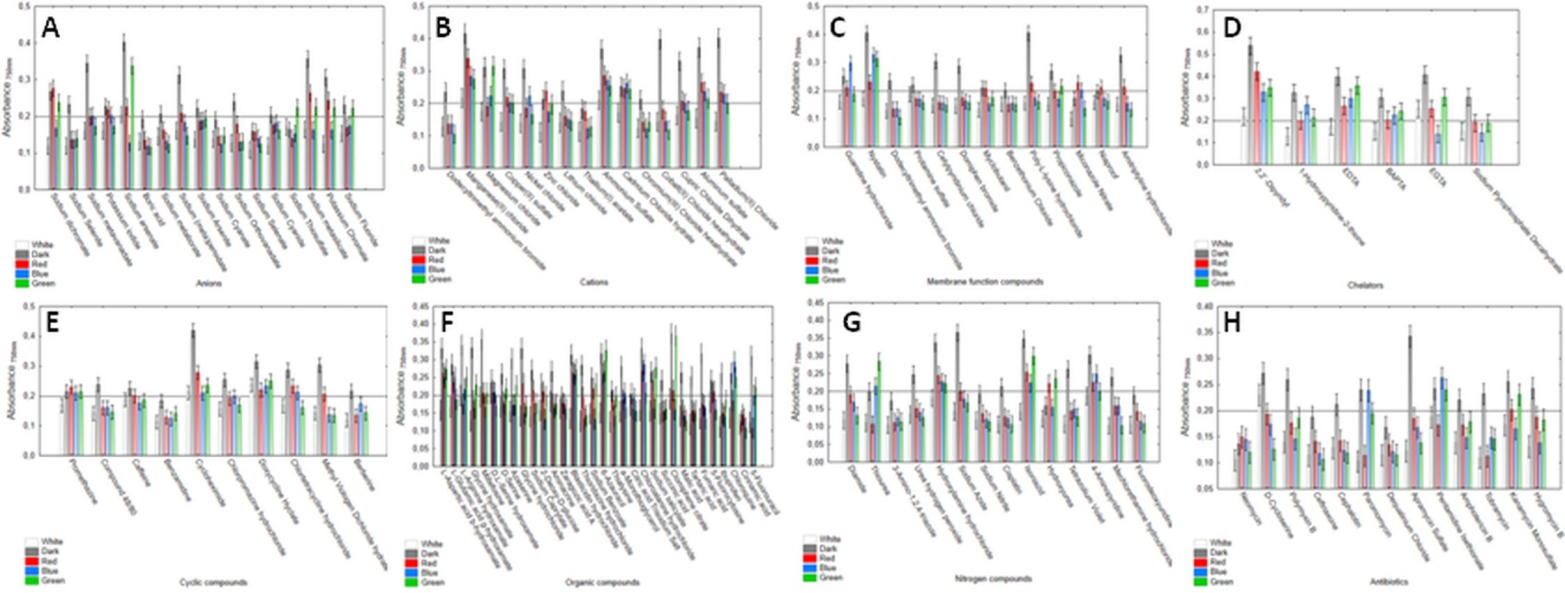
***C*. *unicolor* growth in different lighting treatments (white, red, blue and, green light, darkness) in the presence of A–anions, B–cations, C–membrane function-affecting compounds, D–chelators, E–cyclic compounds, F–organic compounds, G–nitrogen compounds, and H–antibiotics.** The scales represent growth values (OD 750 nm) after 10 days of incubation. The horizontal line at a value of 0.2 represents the growth threshold considered positive. Vertical bars denote 0.95 confidence intervals.

Next, Biolog PM21-PM25 panels were applied to verify the *C*. *unicolor* sensitivity to various chemical compounds in relation to the culture lighting treatments. In general, the rate of growth inhibition of *C*. *unicolor* increased with the dose of the chemical agents. As demonstrated above, the fungus achieved the greatest capacity to utilize a broad spectrum of carbon sources during its growth in darkness. Similarly, *C*. *unicolor* cultivation in the darkness resulted in tolerance to all the tested chemicals (Fig 6). On the contrary, the cultivation in the white light conditions resulted in the strongest sensitivity to the chemical compounds applied (Fig 6). Among the eight tested groups of potentially toxic compounds, *C*. *unicolor* growth was mostly inhibited by antibiotics and nitrogen compounds and was the least potently affected by chelators (Fig 6). However, the inhibitory effect of these chemicals should be considered separately for each substance and light conditions (Fig 7).

Of the 17 examined potentially toxic anions, borate, cyanate, and selenate completely inhibited the growth of the FCL139 strain in all the tested lighting treatments (Fig 7A). In turn, *C*. *unicolor* was the most resistant to arsenate and metasilicate (except for blue as well as blue and white culture treatments, respectively). Surprisingly, the fungus growth in the blue light condition was very sensitive to all anions. In turn, in the green and red lighting conditions, the fungus displayed a moderate anion sensitivity profile. However, *C*. *unicolor* grown in green light showed increased tolerance to sodium arsenate, which was comparable with the effect observed for darkness. *C*. *unicolor* was generally less chemically sensitive to cations than to anions (Fig 7B). The strongest growth inhibition was caused by thallium (I) acetate, chromium (III) chloride hexahydrate, and dodecyltrimethyl ammonium bromide, whereas Mn^2+^ and NH_4_^+^ had no inhibitory effect regardless of the cultivation treatment. It is worth noticing that the fungus cultivated in darkness tolerated well the presence of Co^2+^, Mg^2+^, Cu^2+^, Pd^2+^, and Al^3+^. The presence of most membrane function–affecting compounds resulted in a very intensive inhibitory effect on fungus growth (10 out of the 13 tested chemical agents caused total growth inhibition in most of the culture conditions) (Fig 7C). Again, only in darkness, the fungus was able to grow in the presence of poly-L-lysine hydrochloride, cetylpyridinum chloride, domiphen bromide, and amitriptyline hydrochloride. Moreover, *C*. *unicolor* displayed strong resistance to nystatin in almost all the culture lighting conditions. In contrast to the membrane function–affecting compounds, chelating agents influenced the fungus growth to the least extent. Strong growth inhibition was observed only for sodium pyrophosphate decahydrate; however, the fungus growing in darkness was insensitive to this chelator (Fig 7D). Cyclic compounds constituted a group of chemicals that tended to inhibit *C*. *unicolor* growth (Fig 7E). As previously, in darkness, the fungus was more resistant to cyclic compounds; however, in the presence of promethazine, compound 48/80, caffeine, and berberine the fungus grew very weakly, while benzamidine totally abolished its growth (this chemical strongly inhibited growth in the other tested light culture treatments as well). It is worth emphasizing that *C*. *unicolor* growth was less sensitive (especially in the dark condition) to cycloheximide and doxycycline. Organic compounds constituted the most abundant group of potentially toxic chemical agents tested in this study, and exerted diverse effects on *C*. *unicolor* growth (Fig 7F). Five of the 32 organic compounds (namely: L-aspartic acid, blasticidin, 6-azauracil, chloroalanine, and clomiphene) did not affect the growth of *C*. *unicolor* in all the tested culture conditions except for the white light. Noteworthy, the white light culturing resulted in fungus growth inhibition in the presence of all the tested organic compounds. On the other hand, only cinnamic acid caused strong inhibition of fungus growth in all the tested culture treatments. Most antibiotics and nitrogen compounds (84% and 71% of the total number of tested, respectively) strongly influenced the growth of *C*. *unicolor*. 3-amino-1,2,4-triazole, sodium nitrate, cisplatin, and fluorodeoxyuridine (nitrogen substances) as well as neomycin, ceftriaxone, and dequalinium chloride (antibiotics) exerted a very strong inhibitory effect regardless of the culture conditions (Fig 7G and 7H, respectively). As shown previously, white light made the fungus more sensitive to all nitrogen compounds and almost all antibiotics (12 of the 13 tested compounds); the growth was considered as negative. The white light treatment did not affect the *C*. *unicolor* FCL139 growth in the presence of D-cycloserine (Fig 7H). Moreover, increased resistance of *C*. *unicolor* cultured in darkness to hydroxylamine, azide, isoniazid, and apramycin was evident (Fig 7G and 7H).

## Discussion

Fungal metabolic traits are becoming increasingly important in their identification and taxonomy [27–29]. Technologies based on phenotyping arrays and microplates ensure the most comprehensive metabolic profiling of organisms, offering characteristics of species, individual strains, and entire ecological groups. The combination of the data into a biochemical map can also detect pathways or enzymatic steps unique for a particular strain, species, or genus, which may have potential commercial use [30].

The wood decaying basidiomycete *C*. *unicolor* has previously been widely described as an invaluable source of various biotechnologically applicable compounds [10, 16]. However, no efforts have been made to explore the metabolic capabilities of this fungus and its specific nutritional requirements. Moreover, since *C*. *unicolor* belongs to the group of white rot fungi, comparison of its metabolic characteristics with those of other fungi representing different rot lifestyles could allow further ecological studies of this fungus. The knowledge of changes in its metabolism induced by basic physicochemical factors could be fundamental to study the interactions with the environment and plant material which the fungus may colonize.

We used FF MicroPlates to compare the nutritional preferences and functional diversity of *C*. *unicolor* FCL139 and other fungi representing different rot lifestyles and colonization abilities. Their substrate utilization profiles demonstrated significant metabolic differences between the analyzed strains. In comparison to the other analyzed saprotrophic white rot fungi, *Cerrena unicolor* FCL139 demonstrated an average ability to utilize carbon sources but was capable of metabolizing certain groups of compounds, similarly to *T*. *versicolor* and *P*. *radiata*, which are extensive metabolizers [31]. Carbohydrates were used most universally by all strains, which is not surprising since lignocellulosic material is a major natural substrate for various wood degrading fungi [32]. On the other hand, organic acids, and therefore carboxylic acids (poorly utilized in our studies), are important in maintaining the charge balance in filamentous fungi, well known for their potential to accumulate organic acids in the medium, which does not constitute a direct source of metabolic energy for these microorganisms [33]. *C*. *unicolor* catabolized salicin and arbutin efficiently, which may indicate elevated carbohydrate uptake and metabolism. Additionally, salicin utilization suggests production of enzymes involved in biosynthesis of phenylpropanoids (the starting compounds for biosynthesis of lignin), which constitute the key electron carriers in the fungal wood degradation process [31]. Inducing effect of salicin on endoglucanase synthesis in *Rhizopus oryzae* has also been proved [34].

Next, using PM1 and PM2, a detailed analysis of low weight carbon and energy substrates utilization abilities of *C*. *unicolor* precultured on various sawdust media was performed. PMs have already been successfully used for characterization of the functional diversity and ecology of many fungal species [2]. Interestingly, the fungus exhibited the highest catabolic activity after precultivation on the birch sawdust medium, whereas the application of the mineral LH medium resulted in the weakest fungal growth and metabolic activity. It can be hypothesized that, in comparison to the synthetic mineral medium, *C*. *unicolor* precultivation on birch sawdust triggers the synthesis of enzymes and activation of metabolic pathways responsible for lignocellulose degradation. Birch is one of the several natural habitat occupied by *C*. *unicolor* [6]. Bearing this in mind, it seems not surprising that polymers and carbohydrates constituted groups of carbon sources that were most efficiently and universally metabolized by the fungus, because they mimic natural conditions encountered by *C*. *unicolor* during its saprotrophic lifestyle. Moreover, pectin was proved to be a universal polymeric C-source for fungus growth. In case of brown rot fungi, production of oxalic acid and pectinase during the decay process have been shown to be important in initiation of lignocellulose metabolism and spreading of fungal hyphae through wood [35]. On the other hand, *C*. *unicolor* precultivated on the ash sawdust medium exhibited preferences for utilization of fatty acids. Expression of genes encoding enzymes responsible for fatty acids degradation was proved again to play an important role in the wood colonization by another white rot fungus–*Phlebiopsis gigantea* [36].The capability of some fungal species of utilization of different carbon sources and diversified enzyme synthesis has already been proposed as a mechanism of slow adaptation of higher white rot fungi to changing environmental factors [37].

Knowing that sunlight is a very important abiotic signal for living cells, serving as either a source of energy or information from the surrounding environment and being crucial for survival in nature [4, 5], a comprehensive and comparative phenotypic study of *C*. *unicolor* FCL139 was performed to investigate the effect of variable light conditions on the fungus metabolism and chemical sensitivity. The focus was on metabolic differences observed in applied light conditions. Studies based on the PM system aiming to evaluate the influence of habitat conditions and environmental stresses on the physiological diversity of fungi have been performed [2, 38, 39]. However, phenotypic methods have not been previously applied for photobiological studies in higher fungi. The influence of light on carbon source utilization has been demonstrated repeatedly in filamentous fungi and molds [40].

Pilot studies showing the impact of different wavelengths on the synthesis of several enzymes in *C*. *unicolor* have been performed [41]. The metabolic response is influenced by the carbon source the fungus grows on. It has been shown that light has different effect on fungal growth depending on optimization of the nutritional conditions [5]. In this work, we have shown variable and light-dependent substrate utilization profiles of *C*. *unicolor* cultivated in white, blue, red, and green light as well in darkness. It should be noted that during the measurements additional light pulses were introduced to the fungal cultures. However, the aim of the experiment was to investigate the influence of continuous (constant) light conditions on fungus metabolism based on comparative approach and appropriate controls were applied. The fungus cultured in darkness showed the highest catabolic activity, while in the white culture condition, it was able to assimilate only 40% of the tested carbon sources. In general, efficient growth of the fungus was observed on (i) carbohydrates and (ii) polymers, which are metabolically related to cellulose and hemicellulose and are degraded by enzymes that can break down the complex of polysaccharides and lignin in the plant cell wall [31], as well as (iii) carboxylic acids, which accumulate as a result of polysaccharides metabolism [1, 32] (Fig 4C). In all the culture treatments, *p*-hydroxyphenylacetic acid, found in pathways related to degradation of xenobiotics, was the most readily metabolized compound. A similar observation related to the light-controlled cellulases expression has been made in *Trichoderma reesei*. Light-dependent gene regulation at the level of transcription in a positive feedback cycle in darkness and a negative feedback in white light has been shown, indicating a cellular sensing and response mechanism for the production of these enzymes in *T*. *reesei* [42]. Interestingly, when growing in darkness, *C*. *unicolor* was not able to utilize D-glucose and D-xylose, which were efficiently metabolized in other light conditions. In filamentous *Aspergillus ornatus*, glucose and amino acid uptake was significantly decreased upon cultivation in light prior to production of conidia [43]. The result related to amino acid uptake is also consistent with the data obtained for *C*. *unicolor*. Investigation of the growth patterns of *Trichoderma atroviride* (*Hypocrea atroviridis*) has confirmed that light affected the carbon source utilization and suggested a carbon source-dependent cross-talk between photostimulation, intracellular cAMP levels, and oxidative stress response. A major role of a blue light receptor BLR-1 protein in this regulatory mechanism has been proposed [44].

The results of the chemical sensitivity analysis of *C*. *unicolor* in the different light conditions seemed to correlate well with the metabolic profiles obtained. The greatest tolerance to chemical compounds was observed when the fungus was grown in darkness, while its white-light cultivation caused the strongest sensitivity to the tested chemicals and fungal growth inhibition, which suggests that light conditions can affect *C*. *unicolor* colonization properties. It has already been demonstrated that visible light can be deleterious to microorganisms, e.g. by destroying cytochromes and thus affecting cellular respiration or by producing reactive oxygen species (ROS) that cause damage to DNA, membranes, and other cellular components [45].

The examination of the different groups of potentially toxic compounds showed that *C*. *unicolor* was the least affected by chelating agents. This finding is not surprising if we consider the crucial role of the non-enzymatic oxidative mechanisms involved in wood biodegradation. These low molecular compounds with metal-chelating capability produced during fungus growth catalyze the initial oxidation and depolymerization of wood cell walls during the decay process [31, 46]. Additionally, bearing in mind that Cu^2+^ and Mn^2+^ divalent cations are crucial structural features of laccase and manganese peroxidase active sites [10, 12, 13], the tolerance of *C*. *unicolor* to the presence of these cations in the growth environment seems to be a consequence of the natural fungus lifestyle. Moreover, regulation of the biosynthesis of extracellular lignolytic and cellulolytic enzymes by heavy metals has already been proved. During the degradation of lignocellulose and xenobiotics by white-rot fungi, heavy metals present in the growth environment interfere with both the activity of extracellular enzymes involved in this process and colonization of plant material [47]. In all the light conditions (except the darkness), *C*. *unicolor* growth was sensitive to antifungal antibiotics and nitrogen compounds, which in general have a negative effect on the fungal enzymatic machinery and are considered toxic [48]. As demonstrated in this study, especially white light made the fungus growth sensitive to all of those compounds.

Summarizing, we have performed comprehensive analysis of the metabolic capabilities and chemical sensitivities of *C*. *unicolor* in different conditions, providing information on the global phenotypes and specific nutrient utilization profiles of the fungus. The obtained picture of *C*. *unicolor* metabolism is very complex and the fungus abilities to decompose woody plant material are dependent on various factors. A correlation of fungus substrate utilization profiles, chemical sensitivity, and light culture conditions has been demonstrated. Nevertheless, taking into consideration the complexity of the observed metabolic pathways, these data should be considered as a starting point for further investigations. Coupled transcriptomic and proteomic analysis are still required to demonstrate the involvement of the specific proteins in fungal metabolic pathways and to better understand the role of *C*. *unicolor* in the environment.

## Supporting information

S1 Dataset (OD data). **Comparative analysis of the fungal metabolic profile using BIOLOG FF MPs**.
(XLSX)

S2 Dataset (OD data). **Evaluation of the effect of *Cerrena unicolor* growth on various sawdust media using BIOLOG PM1-2 PMs**.
(XLSX)

S3 Dataset (OD data). **Evaluation of the effect of light conditions on *Cerrena unicolor* metabolic profiles using BIOLOG FF MPs**.
(XLSX)

S4 Dataset (OD data). **Evaluation of the effect of light conditions on *Cerrena unicolor* chemical sensitivity using BIOLOG PM21-25 PMs**.
(XLSX)
